# Cancer Incidence of Finnish Sami in the Light of Exposure to Radioactive Fallout

**DOI:** 10.3390/ijerph18158186

**Published:** 2021-08-02

**Authors:** Leena Soininen, Helena Mussalo-Rauhamaa

**Affiliations:** 1 Arctic Centre, 96101 Rovaniemi, Finland; leena.soininen@fimnet.fi; 2 Institute of Clinicum, Department of Public Health, University of Helsinki, 00014 Helsinki, Finland

**Keywords:** Finnish Sami, radioactive fallout, cancer risk

## Abstract

This article summarizes the results of studies on the exposure of the Finnish Sami people to radioactive fallout and the estimations of the related cancer risk. We also discuss the lifestyle, genetic origin and diet of this population. The Sami people are an indigenous people who live in the northern part of Scandinavia and Finland. The review is based on the available scientific literature of Finnish Sami. The traditional Sami diet, high in animal products, persists in Sami groups still involved in reindeer-herding, but others have adopted the typical diet of western cultures. Studies have consistently shown an overall reduced cancer risk among the Finnish Sami people, except for stomach cancer among the Skolt Sami. Common cancers among the Finnish main population, such as prostate, breast and skin cancer are especially rare among the Finnish Sami. The incidence of cancer among the Finnish Sami are mostly similar to those of the Swedish and Norwegian Sami. To conclude, we observed no effect of radioactive pollution on cancer incidence. The lifestyles and environments of the Sami are changing, and their cancer mortality rate today is similar to that of the majority of the Finnish and western population.

## 1. Introduction

**Figure 1:**
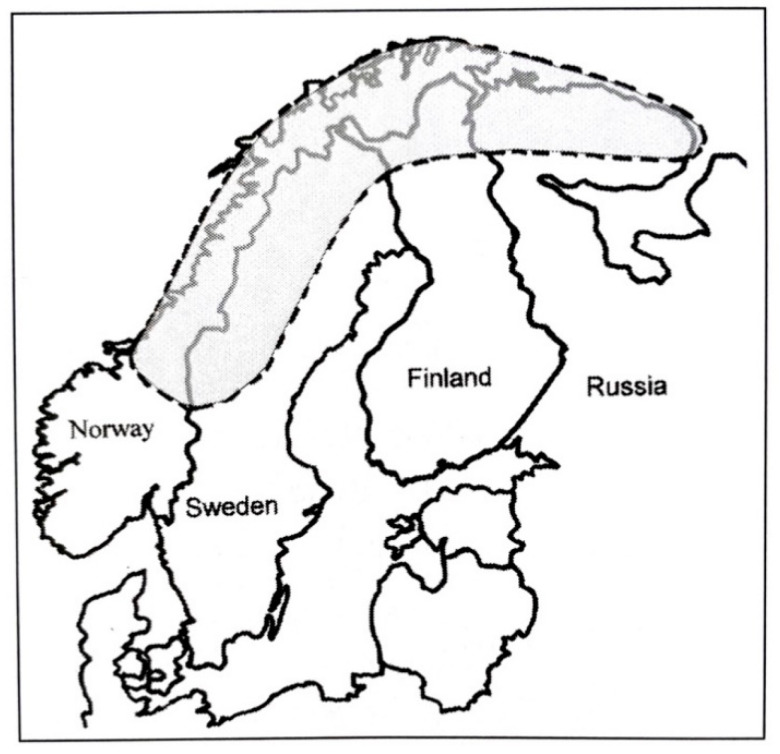
Area of Sami population.

The traditional Sami area extends from the northern parts of the Kola Peninsula in Russia to the north of Finland, Norway and Sweden. (Figure 1) The total number of Sami is estimated to be 75,000–100,000. Most Sami live in Norway (50,000–65,000) and Sweden (about 20,000–40,000). Finland has about 9000 Sami [1]. Already in 2010, most of the Finnish Sami, 60%, lived outside the Sami area. The Finnish Sami consist of three different Sami subgroups—the North Sami, Inari Sami and Skolt Sami—who today have mixed with each other and with Finns.

In the circumpolar arctic and subarctic latitudes, the Sami peoples’ economy was based on reindeer and caribou herding and fishing. During the winter months, in Finnish Lapland, reindeers fed solely on reindeer lichen (*Cladonia*), which they could reach through the snow. Lichen grows very slowly and its prothallium has a unique capacity to retain and absorb nutrients and elements. It also effectively retains any radionuclides present in global fallout, both man-made and natural. Radionuclides also accumulate in terrestrial ecosystems and water bodies, and the rates of decline in contamination levels in biota in both types of ecosystems are slower than those in the atmosphere. In general, radionuclide contamination levels in terrestrial biota have consistently been higher than those in marine biota. In the terrestrial environment, the highest activity concentrations have been found in products harvested from natural or semi-natural ecosystems [2].

The two major sources of radioactive fallout in the Arctic region have been nuclear weapon testing and the Chernobyl accident. Between 1960 and 1991, the former Soviet Union also dumped radioactive waste in the Kara and Barents Seas. These dumped wastes were both liquid and solid, the latter including reactor compartments and entire submarines. The dumping of radioactive waste on the northern shore of the Kola peninsula is also a risk source [2]. 

### 1.1. Sources of Artificial Radionuclides 

Since the first nuclear weapon test in 1945, more than 2000 nuclear explosions have taken place of which over 500 were carried out in the atmosphere. Nuclear tests can be divided into three phases based on the fission and induced radioactive products released: viz. 1945–1958, 1961–1962 and post–1963. In the first phase, only about 20–30% of the explosions entered the stratosphere. In the second phase, nearly all of the reported atmospheric explosions entered the stratosphere. From the underground explosions, only small amounts of radioactive products leaked into the atmosphere. Of the nuclear test explosions in September 1957 to December 1962, 88 took place in the Arctic on the island of Novaya Zemlya and 85 of these were atmospheric tests. At the end of 1962, the USA, the USSR and Great Britain ceased atmospheric testing. After the Partial Test-Ban-Treaty of 1963, nuclear tests have still been carried out by France, the People’s Republic of China, India, Pakistan and North Korea [3,4]. 

Particles can float in the stratosphere for very long periods of time. They spread themselves apart horizontally, so that eventually, they can be found throughout the stratosphere. In polar regions, during the winter and early spring, vertical mixing brings material down into the troposphere, which is then cleansed by precipitation. Radionuclide activity concentrations in the air and precipitation have closely reflected the rates of emission of radionuclides into the atmosphere from above-ground nuclear weapon tests. Peaks were identifiable during the period when most tests were conducted, or were associated with specific events, such as vented underground nuclear tests or accidental releases [2].

### 1.2. Radionuclides in the Air Due to Fallout 

As a result of the global fallout from the atmospheric nuclear weapon tests performed in the northern hemisphere, the majority of radio cesium (^137^Cs) deposition occurred during the period 1955–1966. Precipitation and latitude have been identified as the principle factors determining spatial variation in global fallout [2]. The amount of fallout varies according to the season, and is at its highest in the spring, from April to May, and at its lowest in November. Factors such as transport mechanisms in the atmosphere and the magnitude of rainfall affect the geographical and seasonal variations of fallout [5,6].

The Finnish Meteorological Institute collected aerosol samples from the Artic Circle during 1965–2011 onto filters at its Rovaniemi monitoring station in Southern Lapland in April. The activity concentration of ^137^Cs in the surface air of Rovaniemi was high in 1965 (320 ± 3 µBq/m^3^), but even higher in April–June 1986 (1294 ± 7 µBq/m^3^) [7]. The varying results of ^137^Cs concentration levels are in accordance with studies in the Northern Hemisphere, where the atmospheric activity concentration peaks occurred in 1963 (deposition maximum from atmospheric nuclear weapon testing) and in 1986 (Chernobyl accident) [8,9]. Volatile radionuclides such as ^137^Cs have been transported from the destroyed Chernobyl reactor to Finnish Lapland [7]. 

Based on the filter samples of the Rovaniemi monitoring stations, the activity concentration of ^90^Sr was at its highest in 1965 (145 ± 30 µBq/m^3^). The atmospheric concentration of ^90^Sr has been constantly decreasing in Rovaniemi since 1965. After April–June 1968, the activity concentration of ^90^Sr was under limit of detection. Total beta radioactivity in the surface air of Rovaniemi was at its lowest value of 140 µBq/m^3^ in July–December 1986 and at its highest value of 7410 µBq/m^3^ in April–June 1986. Intermediate radionuclides such as ^90^Sr from the destroyed Chernobyl reactor have not reached this region in Finnish Lapland [7].

The activity concentrations of ^238^Pu, ^239+240^Pu and ^241^Am were also determined from the air filters collected in the Rovaniemi monitoring station in 1965–2011. The main sources of plutonium were the atmospheric nuclear weapon tests in the 1950s and 1960s. The Chernobyl accident in 1986 only had a minor effect on air concentration. The major source of Pu-238 in the stratosphere was the burn-up of the SNAP-9A satellite over the South Pacific Ocean in 1964 [10,11,12]. 

### 1.3. Food Chain Studies

The ability of lichen to accumulate radionuclides was revealed in 1958 and led to profound studies of the lichen–reindeer–man food chain. ^137^Cs was the radionuclide that most abundantly accumulated in reindeer meat-consuming populations; in Finland among the reindeer-herding Lapps. Other studied radionuclides that originated from nuclear tests were ^90^Sr, ^55^Fe, ^14^C and Pu-isotopes 239, 240 and 238.

Investigations of the arctic food chains started in Finland in 1959 [13]. Three notable food chains have been revealed: surface water–sedges and horsetails–cow’s meat and milk; surface water–plankton–fish–man; and lichen–reindeer–man. Several radionuclides were found in Finnish Lapland: ^137^Cs and ^90^Sr are long living and considerably significant contaminants of food and the human body. ^137^Cs behaves like potassium and accumulates in the muscles of reindeer and through them, in humans. Strontium behaves like calcium and accumulates in the bones of reindeers. Lead and polonium are also stored in bones. This is why the lichen–reindeer–man food chain has proven to be the most important for reindeer-herding populations. Ecological factors, and not a high fallout level, accentuate the enrichment of ^137^Cs through these food chains. Low potassium content, the harsh climate, a low number of species, and high dependence on a few food species has led to the enrichment of cesium [14,15,16,17].

The Department of Radiochemistry at the University of Helsinki has been measuring the ^137^Cs contents of lichen, reindeer and Lapps since the beginning of the 1960s [15,16,17]. The ^90^Sr concentrations in Lapps were also studied in the 1960s [18]. In the same decade, the behavior of natural radionuclides ^210^Po and ^210^Pb along the food chain was described by Kauranen et al. [19,20]. Later, in the 1970s, the ^239,240^ Pu content of lichen, reindeer and the organs of five Lapps were determined [21,22,23]. Recently, a thorough review of the transuranium elements along the lichen–reindeer–man food chain in Finnish Lapland was published [11]. 

About 25% of the ^137^Cs contained in lichens is absorbed by reindeer [24]. As a result, Finnish Lapps carry about 50 times the ^137^Cs burden of Finns in the southern part of the country. The amount of radioactive cesium (^137^Cs) in the reindeer meat in Finland was comparable to the amounts in the reindeer meat of other arctic areas (Table 1). 

### 1.4. Studies of Sami People

In 1962, a systematic whole-body measurement of a group of reindeer-herders began in the northernmost part of Finnish Lapland, in Inari and Utsjoki. The Department of Radiochemistry of the University of Helsinki annually measured the radiocesium activity of 80–100 Sami until 1977. 

**Figure 2:**
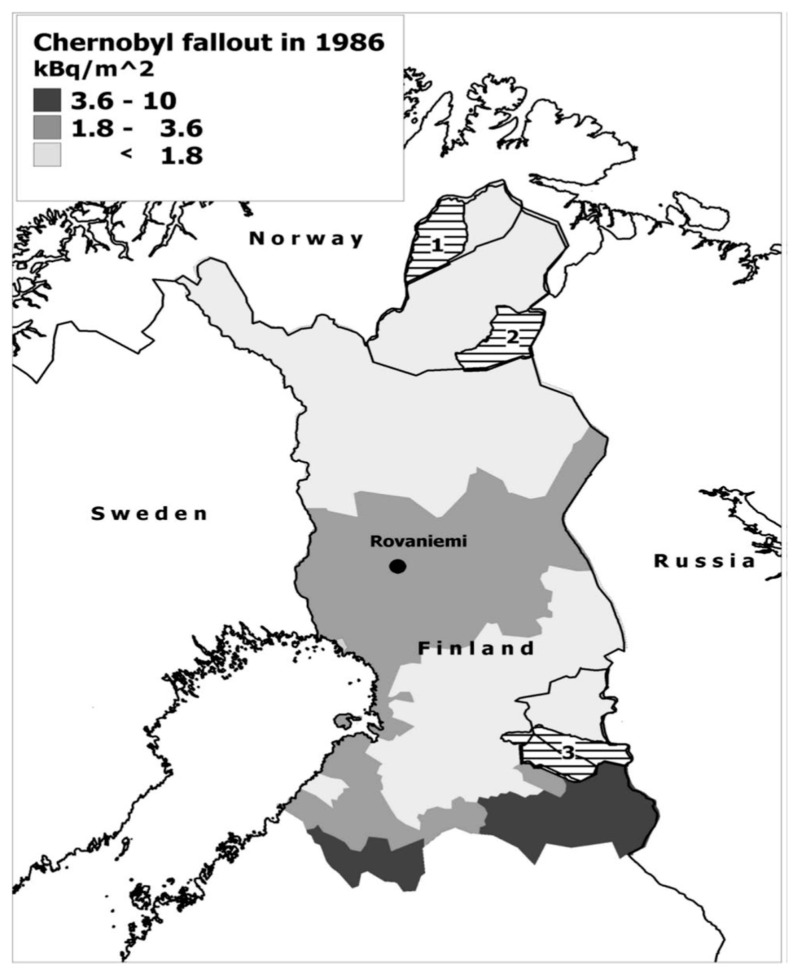
Chernobyl ^137^Cs fallout in Northern Finland. The reindeer-herding cooperatives: (1) Paistunturi, (2) Ivalo ja (3) Halla. The borders of the municipalities of Utsjoki, Inari, Suomussalmi and Hyrynsalmi are also shown [28].

The next measurements were not carried out until 1986, three weeks before the Chernobyl accident [17,26,27,28]. These measurements were taken by two mobile whole-body counters: one from the Radiation and Nuclear Safety Authority (STUK) and one from the Department/ Laboratory of Radiochemistry, University of Helsinki. In 1987–1994, measurements were also carried out in the Halla cooperative, which is the southernmost reindeer-herding cooperative in Finland (Figure 2). Reindeer-herding, however, is not tied to the Halla peoples’ identity [28]. These whole-body measurements were continued until 2005. 

**Figure 3:**
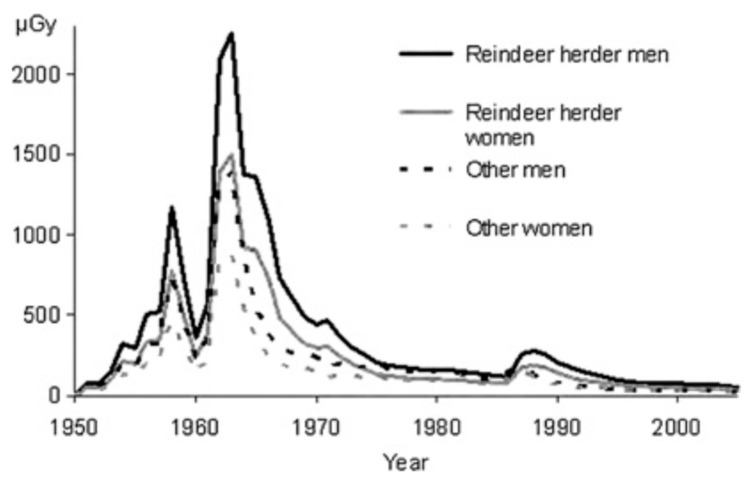
^137^Cs body burden in Inari population from 1965 to 2013 [29]. After the mid-1960s, ^137^Cs activity concentrations in reindeer meat decreased, and an effective ecological half-life of about 5–10 years was observed until the Chernobyl accident in 1986.

The whole-body measurements of the gamma radiation in people revealed that the mean ^137^Cs body burden in Finnish reindeer-herders in the Inari population reached its maximum value of 55,000 Bq in 1965 [17]. After this, it started to decrease. In April 1986, it was 5000 Bq in Finnish reindeer-herders and 3200 Bq in all Sami (Figure 3). 

Studies from 1987–1989 show that the removal of Chernobyl-derived cesium varied in different parts of the Finnish reindeer management area. The effective half-lives of the ^137^Cs body burden among male and female reindeer-herders in Northern Finland were 5.5 ± 1.3 and 4.4 ± 0.9 years, respectively. In the Halla reindeer-herding cooperative, the effective half-lives in reindeer-herders were about one to two years shorter [28].

A study carried out by Leppänen et al. [28] also indicated that the removal of cesium from the Chernobyl fallout in the reindeer–man food chain was quicker than that of the cesium originating from global fallout which was also supported by the longer effective half-life of cesium in reindeer meat. The effective half-life of cesium in reindeer meat during the 1960s and 1970s was about four years longer than that in the post-Chernobyl era. However, the habits of reindeer-herding are now changing: lichen no longer plays such an important role and today, reindeer management is increasingly dependent on summer fodder (grass, weeds, leaves). 

^137^Cs, ^90^Sr, ^210^Pb, ^210^Po, ^239,240^Pu and ^238^ Pu activities have been found in the lung, liver, muscle and bone samples of male Sami [30,31] (Table 2). The ^137^ Cs concentration in the liver of the Sami was 40 times higher than that in liver of southern Finns. The ratio of ^210^Pb concentration in the liver of the Sami to that in southern Finns was 1.8. For ^210^Po, the corresponding ratio was 5.6. For comparison, in 1967, the ratio of ^210^Pb and ^210^Po concentrations in the placentas of Lapps to those of southern Finns were 2.3 and 12.4, respectively [19]. The plutonium concentrations in the tissues of male reindeer-herders and male Southern Finns did not differ (Table 2). 

The ^137^Cs concentrations in the lungs of the Sami and southern Finns differed as dramatically as those found in their liver. The ^210^Pb content of the lungs was about the same in both population groups but the ^210^Po content in the Sami was about twice that in the southern Finns. The ^210^Pb and ^210^Po concentrations in the bones of the Sami did not differ from those of the southern Finns. The average ^90^Sr concentration in the bones of the Sami was about twice that in the southern Finns. The fallout of ^90^Sr is mainly concentrated in the bones of reindeer, creating a break in the transfer of ^90^Sr along the lichen–reindeer–man food chain.

### 1.5. AMAP Studies

**Figure 4:**
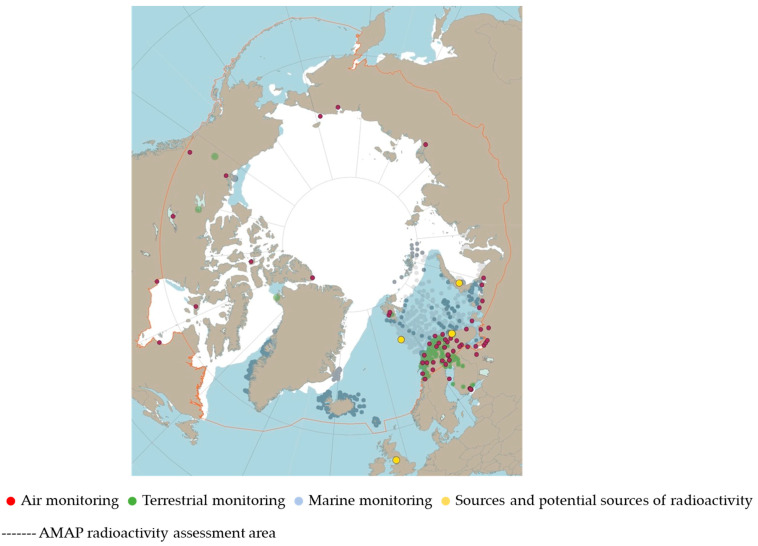
Monitoring results and sources and potential sources of radioactivity discussed in AMAP assessment 2015 [32].

When concern about the arctic environment increased, all eight arctic countries (Canada, Denmark, Finland, Island, Norway, Russia, Sweden and the USA) established a common monitoring project in 1991 called the Arctic Monitoring and Assessment Project (AMAP) (Figure 4) [32]. “Human Health” was also assessed by subprojects of AMAP. The group targeted by the AMAP human health project was expecting mothers and their neonates. A newborn is considered the final destination of soluble environmental toxins in the food chain, as these toxins transfer into breast milk from the mother’s fat tissue [33]. The Finnish AMAP Human Health targeted mercury, lead, cadmium, copper, zinc, selenium, 15 different polychlorinated biphenyl compounds or PCB congeners and 11 different pesticides [34]. 

At the end of the 1980s, the pollution from the Russian smelters in Nikel and Montshegorsk on the eastern side of Lapland came to light. Their atmospheric emissions included mainly sulphur, copper and nickel. In addition, in the emissions there were several other metals [35].

Since the beginning of the 1990s, studies of the concentration of carcinogenic substances and radioactivity in the Sami and other northern residents have been published. Many of these studies have concerned mercury, because high values of the element have been found in the food chain: soil–artificial lakes—fish—man. Other metals such as cadmium, copper, zinc, nickel, arsenic, lead, chrome and selenium have also been examined [34,36,37,38,39,40].

The selenium content of the Lappish people was studied in the 1980s and 1990s because of its protective function against increased mercury and other toxicants [34,39,41]. It has also been found that low serum selenium levels in men are associated with an increased risk of cancer, the strongest association observed being for cancers of the stomach and lungs [42].

Environmental researchers [35] have shown that the trans-boundary pollution originating from the Kola region has levelled out and has not been significant enough to cause elevated pollution concentrations in the Lappish people. 

## 2. Health and Cancer Risk of Sami People

Today, the health risks of the Sami in terms of lifestyle parameters (nutrition, physical activity, smoking and drinking habits), metabolic parameters (weight), and blood pressure do not differ very much from those of the general Finnish population.

### 2.1. Nutrition

It has been said that there has never been famine in Lapland, as the Sami have always had reindeer meat, fish and berries. These have now become supplemented by cereal products, vegetables, fats and dairy products (Table 3).

**Figure 5:**
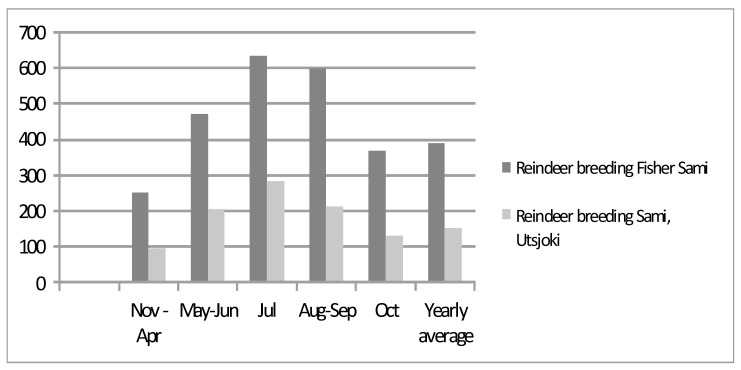
Consumption of fish (grams per person per day) by reindeer-herding Sami men in Utsjoki and reindeer-herding Fisher Sami men in Inari in the 1960s in different months in the individual survey [25].

Fish has always been important food for every Sami group. The reindeer-breeding Fisher Sami (Inari Sami) used the most the fish year-round (Figure 5). The Sami traditionally caught whitefish, trout and graylings. Today, however, they can buy the same foodstuffs as in the southern parts of Finland. 

### 2.2. Smoking and Alcohol

Smoking is more common among reindeer-herders than among the general population. In 1986, a health study of reindeer-herders in Finland showed that 33.8% of the reindeer-herding Sami smoked. The corresponding non-Sami figure was 27.4% [43].

The intake of alcohol in the Inari-Utsjoki area was 14.7 g/day [44]. The alcohol consumption of Sami women is not known.

### 2.3. Exercise

Before the arrival of snowmobiles, reindeer-herding was very demanding exercise. However, operating a snowmobile also requires good physical fitness; when the vehicle gets stuck in the snow, a great deal of power and very healthy back is needed to get it moving. This means that active reindeer-herders still exercise a great deal when they drive in the woods or outside the trails. Näyhä et al. [45] sent a postal interview to reindeer-herders and found that snowmobile operation is highly strenuous and causes problems in the limbs and the back.

### 2.4. Blood Pressure

A study of Sundberg et al. [46] showed that systolic pressure rose more with age among women than among men. In neither sex did age affect diastolic pressure. The finding that the diastolic blood pressure of the Sami had no clear age dependence, as in the Finnish general population, supports the hypothesis that the resting blood pressure level is influenced by different kinds of stress associated with technological development and an urbanized life.

### 2.5. Weight

Näyhä et al. [47] found that Finnish Sami and non-Sami reindeer-herders were overweight, some even obese. The same was found among Swedish Sami [48]. A study by Jokelainen in 1962 [25] found North Sami women were a little overweight. Sami and the Norwegians in Finnmark had a normal weight index [49].

### 2.6. Blood Lipids and Antioxidants

The cholesterol and triglyceride concentrations of 828 Sami were studied. Samples were taken in 1969 and 1970. The serum levels of the concentrations of cholesterol and triglycerides were the same as those in the Finnish rural population [50]. Moreover, no differences were found between the different Sami groups [51]. The difference in the total cholesterol among Swedish Sami and non-Sami was also small [48]. In 1989, the concentrations of serum cholesterol and LDL cholesterol were higher among the Sami reindeer-herders than the non-Sami reindeer-herders. Serum HDL cholesterols did not differ [52].

Luoma et al. [52] also studied the serum alpha-tocopherol, albumin, selenium and cholesterol concentrations of Sami reindeer-herders. Alpha-tocopherol concentration increased with the consumption of reindeer meat, and serum selenium increased with fish consumption. The favorable serum antioxidant status among reindeer-herders was credited to the local diet, and hence there were no differences between the Sami and the non-Sami. The good antioxidant status was also connected to low mortality from cardiovascular diseases, despite high cholesterol concentrations in the blood. 

## 3. Cancer Studies

Some epidemiologic studies have examined cancer among the Finnish Sami [29,53,54]. Cancer mortality was also studied by Soininen et al. [55]. The “additional effect” of low radiation (from the Chernobyl accident) on cancers in Finland was studied by Kurttio et al. [56] and Auvinen et al. [57].

According to Finnish law, ethnic background must not be described in any population statistics, meaning that epidemiological studies must find some way to specify their cohorts. The study group of Soininen et al. [53] identified all the people living in the two northern municipalities of Finland (Inari and Utsjoki) on 31 December 1978 from the National Population Register. The Sami cohort was extracted from this using the updated grouping of Sami from the International Biological Program, Human Health Adaptability study in the 1960s. The cohort members’ dates of death and emigration were obtained from the Population Register. Cancer was followed up from the 1979–1998 [53] and 1979–2010 [54] files of the population-based countrywide Finnish Cancer Registry, using personal identification codes as the key. Cause of death in 1979–2005 was obtained from Statistics Finland [55].

A person representing at least 75% of any ethnic Sami group was classified as Sami. A non-Sami was a person with no Sami ethnicity, and the remaining people were classified into a mixed group. The Sami group was divided into subcategories of North Sami, Inari Sami and Skolt Sami, using the same principle: to be classified into a specific Sami subgroup, a person had to represent at least 75% of one type of the three specific Sami ethnicities. Altogether 2087 people had 75% Sami ethnicity: 1002 were North Sami, 518 were Inari Sami, 392 were Skolt Sami and the mixed group comprised 175 [53,54,55]. 

The cohort of Kurttio et al. [29] consisted of 36,461 Finnish residents (total Finnish Artic population), identifiable from the Population Register Center, and born in the northernmost municipalities of Finland (Inari, Utsjoki, Enontekiö, Sodankylä and Petsamo). The unique personal identity codes of the people in the base cohort and of their children, parents and spouses, as well as their dates of death or emigration and mother tongue were obtained from the Population Register. People registered as reindeer-herders (1239 persons) in the censuses from 1970 to 1995 were identified through Statistics Finland. The total number of people engaged in reindeer-herding was 2786. The number of Sami was 2037, of which 45% were reindeer-herders. In Finland, reindeer-herding is not restricted to the Sami as it is in Sweden.

Annual average internal radiation doses, based on ^137^ Cs whole-body measurements, were assigned by birth year, gender and reindeer-herder status. Incident cancer cases of *a priori* selected cancer types in the study cohort during 1971–2005 were identified from the Finnish Cancer Registry. The internal doses that the Sami and reindeer-herder population have received over the years were calculated later, and their influence on cancer has been estimated [29]. 

The standardized incidence rates (SIR) of cancers were calculated in the study of Soininen et al. [53] and Soininen [54] by using the Finnish population as the reference population. Kurttio et al. [29] used the population of Northern Finland (Oulu Hospital District), Table 4.

The incidence of all cancers among all the Sami in both studies was low. In the study by Soininen et al. [53] the SIR of all cancers of the Sami was 0.64 (95% Confidence Interval 0.53–0.76) and the corresponding SIR of the Non-Sami was 0.93 (95% CI 0.81–1.10). The SIR ratio was 0.69 (0.55–0.88).

In 1971–2005, in the study of Kurttio et. al. [29] the incidence of all cancers was still lower 0.60 (95% CI 0.50–0.71), and later in 1979–2010 in Soininen’s cohort [54] it was 0.69 (95% CI 0.69–0.78). The ratio of SIR for Sami /Non-Sami was 0.77 (95% CI 0.65–0.91). In the study of Kurttio et al. [29] the incidence of all cancers of the total Finnish Arctic population, Sami and Non-Sami was higher, 0.86 (95% CI 0.82–0.89).

For the North Sami in 1979–2010, the SIR of all cancers was 0.68 (95% CI 0.55–0.82) and for the Inari Sami, 0.57 (95% CI 0.43–0.74). The corresponding SIR for the Skolt Sami was 0.96 (95% CI 0.71–1.27), because of their high incidence of stomach cancer, SIR 3.4 (95% CI 1.47–6.69) [54].

The high stomach cancer incidence might be due to something other than radioactivity, such as the lack of important nutrients [25,58]. Before the “refrigerator” days, the Skolts ate dried, salted fish and earlier also putrefied sour fish. Electricity only arrived in Sevettijärvi in 1979. Still in the 1960s and 1970s, the Skolt Sami had fewer reindeer than the other Sami. Their status among the other Sami and Finns was the lowest, and their socioeconomic situation was poor [59]. No fresh fruits or vegetables were available.

The incidence of stomach cancer in the Sami communes (Inari, Utsjoki, Enontekiö) was higher than the general incidence in Finland until 1985 (Finnish Cancer Registry). The SIR of the stomach cancer of reindeer-herder Sami in 1971–2005 was 1.42 (95% CI 0.65–2.68) [29].

Of the other radiation cancers, only the risk of ovarian cancer was significantly increased among the non-Sami, with an SIR of 2.03 (95% CI 1.02–3.64). The risk also seemed to be increased among the North and Inari Sami, although not significantly. During the following decade, 1999–2010, it was lower, the SIR of the Sami being 1.47 (95% CI 0.30–4.30) and that of the non-Sami 1.59 (95% CI 0.64–3.28). The Skolt Sami had no cases of ovarian cancer during the study period 1979–2010 [54].

In the study of Kurttio et al. [29] none of the cancer sites were significantly associated with lifetime cumulative radiation dose. The estimated radiation dose was based on ^137^Cs whole body measurements during 1971–2005. The SIR of the stomach cancer and combined group of radiation-related cancer sites among the Arctic population increased with the cumulative radiation dose received before 15 years of age (*p* = 0.004). However, the authors recommend to interpret the findings with caution.

## 4. Cancer Mortality of the Sami

The follow-up of cancer mortality began on 1 January 1979 and ended at death or on 31 December 2010, whichever came first. The overall cancer mortality among the Finnish Sami in 1979–2010 was statistically significantly lower than the mortality of the general population of Finland, the standardized mortality ratio (SMR) being 0.83 (95% CI 0.70–0.98) [55]. The standard population was the population of Finland (1.00). The corresponding figure for the non-Sami was 1.00 (95% CI 0.87–1.13).

### The Additional Effect of Chernobyl Fallout to Cancer Incidences

The Chernobyl fallout was much smaller in Lapland than all the atom explosions in the atmosphere since 1945. In Finland, most of the Chernobyl fallout affected the southern parts (Figure 2). Kurttio et al. [56] studied whether the incidence of all cancer sites combined was associated with the radiation exposure due to fallout from Chernobyl. The emphasis was on the first decade after the accident, to assess the suggested “additional effect”. The part of the Finnish population (2,095,822 people) with stable residence in the first post-Chernobyl year (between May 1986 and April 1987) was studied. The analyses were based on 250 × 250 m grid squares covering all of Finland and all cancer cases, except breast, prostate and lung cancers. Cancer cases in four exposure areas (from <0.1 mSv to >0.5 mSv) before the Chernobyl accident (1981–1985) and after it (1988–2007) were compared, taking into account cancer incidence trends for the longer period prior to (since 1966) the accident. There were no systematic differences in cancer incidence in terms of radiation exposure. The large, comprehensive cohort analysis of the relatively low levels of the Chernobyl fallout in Finland observed no cancer promotion effect.

Auvinen et al. [57] conducted a similar study of a cohort of about 3.8 million people who had lived for 12 months in the same dwelling following the accident. The exposure categories and follow-up years of total cancer incidences were the same as those in the study by Kurttio et al. [56]. Only in the highest exposure group was an incidence ratio (RR) of 1.11 (95% CI 0.90–1.37) found for female colon cancer. This was an 11% increase from the expected incidence rate for the highest exposure group after the latency period.

## 5. Cancer among the Sami in Other Countries

Wiklund et al. [60] found that the cancer risk of the Swedish Sami (1961–1984) was low. The cohort consisted of 2034 reindeer-breeding Sami and the expected cases were calculated on the basis of the total cancer cases in the Swedish population. The SIR for all cancers was 0.61 (95% CI 0.05–0.75) and for the stomach cancer it was 2.25 (95% CI 1.46–3.32). Cancers of liver and cancers of biliary passages were increased, SIR 1.32 (95% CI 0.49–2.88). Incidences of all other cancers were lower than those among general population in Sweden. During the years 1961–1997, the low cancer incidence of the Swedish Sami did not change [61]. In comparison to the general Swedish population, the SIR of all cancers was still 0.60 (95% CI 0.52–0.69) and in comparison to the geographically matched reference population, it was 0.78 (95% CI 0.57–0.89). When the 1961–2003 incidence was studied again by Hassler et al. [62], an increase of cancer incidences was found. The SIR of all the sites was 0.90 (95% CI 0.85–0.96) among men and 1.04 (95% CI 0.97–1.10) among women. The corresponding figures (1979–2010) of Finnish Sami were among men 0.63 (95% CI 0.51–0.75) and among women 0.77 (95% CI 0.63–0.92) [54] and Norwegian Sami (1970–1997) among men 0.78 (95% CI 0.73–0.84) and among women 0.84 (95% CI 0.78–0.91)] [63].

The change in the lifestyle habits of the Swedish Sami may have been greater than that of the Sami in other countries but it is going on in all countries. Some of the Swedish Sami live further south than the Sami in other countries (Figure 1). The figures for Swedish reindeer-breeding Sami are more similar to those of the Finnish Sami.

Among indigenous people in other Arctic countries cancer has been rare but today this is no longer the case [64].

Skuterud et al. [65] calculated the integrated effective ^137^Cs dose for reindeer-herders in Norway. It was about 18 mSv. This dose represents an insignificant increase in the risk of developing cancer.

## 6. Conclusions

No clear association was found between lifetime cumulative radiation exposure and cancer incidence among the Finnish Sami. The incidences of several cancers were lower among Sami and whole arctic population than among Finnish general population. A weak association was found between radioactive exposition before 15 years of age and stomach cancer. There are several other causes of cancer in addition to radioactivity e.g., smoking, alcohol and other toxic substances, genetic susceptibility and lack of healthy food. Combined effects are also important.

Finding a clear correlation between low radioactive radiation and cancer in such a small population is difficult. AMAP has connected data from Arctic countries, and although some topics can be researched together, culture, ethnic differences and living standards have many confounding factors. Health monitoring and research among the Arctic peoples are needed, especially concerning cancer.

## Figures and Tables

**Table 1:** ^137^Cs contents as Bequerels (Bq/100 g) in reindeer and caribou meat in different countries [25].

Year	Country	^137^Cs
		Bq/100 g Fresh Weight
1959	Norway	28.5
	Finland	74
1960	Norway	130
	Finland	111
	North-eastern Alaska ^1^	18.5
1961	Norway	81
	Sweden	11.1–85
	Finland	66.6
	Greenland	35.5
	North-eastern Alaska ^1^ (30)	26.1
1962	Sweden	85–148
	Finland	23.7–64
	Greenland	111
1963	Sweden	74–93
	Finland	17.8–174
	Greenland	30.3
1964	Finland	222

^1^ Caribou meat.

**Table 2:** Concentrations (Bequerels) of radioactive cesium (^137^Cs), plutonium (^239^**^–^**^240^Pu), lead (^210^Pb), polonium (^210^Po), and strontium (^90^ Sr) in liver, lung and bone tissues (post-mortem samples) in the 1970s [31].

Tissue	^137^Cs	^239^^–240^Pu	^210^Pb	^210^Po	^90^Sr
	Bq	mBq	Bq	Bq	mBq
Liver of Sami	79.9	15.5	0.48	3.2	
Liver of Southern Finns	<1.9	15.2	0.16	0.57	
Lung of Sami	43.7	1.1	0.09	0.30	
Lung of Southern Finns	<1.9	1.2	0.12	0.14	
Bone of Sami	-	1.7	-	-	70
Bone of Southern Finns	-	1.8	1.22	1.07	30

**Table 3:** Percentage contribution of different foodstuffs to total energy in families of different Sami groups [25].

	Nomadic and Settled Reindeer-Breeding and Fisher Sami Groups	Skolt Sami
Foodstuff	%	%
Cereal products	28.9–40.5	39.6–50.1
Potatoes, other vegetables and peas	3.9–5.5	3.1–5.5
Berries and fruit	0.9–1.9	1.0–1.6
Sugar	9.4–11.6	11.3–19.1
Margarine	12.3–16.8	15.5–21.1
Butter	0.2–5.1	0.5–2.3
Other milk products	8.8–29.5	4.4–5.8
Meat and meat products	3.0–14.6	3.5–11.4
Fish	2.3–7.1	1.2–3.3
Intake of energy, kcal	2755–3395	2595–2660

**Table 4:** Observed (Obs.) and expected (Exp.) number of cancer cases and standardized Incidence ratios (SIR) with 95% confidence intervals (CI) among Sami and Non-Sami population.

	Soininen et al. 2002 [53]								Kurttio et al. 2010 [29]							
	Years 1979–1998								Years 1971–2005							
Reference Group	Finnish Total Population								Population of the Region of Oulu University Hospital							
	Sami				Non-Sami				Sami				Non-Sami			
Site	Obs	Exp.	SIR	95% CI	Obs.	Exp.	SIR	95% CI	Obs	Exp.	SIR	95% CI	Obs.	Exp.	SIR	95% CI
Size of population	2100				4174				2037				32,616			
person years	37,415				77,707				2299				821,094			
All	111	173	0.63	0.33–0.76	226	244	0.93	0.81–1.0	140	234	0.60	0.50–0.71	2490	2839	0.88	0.84–0.91
Thyroid	1	2.4	0.41	0.01–2.3	4	4.5	0.89	0.24–2.3	2	4	0.49	0.06–1.77	66	67	0.99	0.77–1.25
Leukaemia	2	4.1	0.49	0.06–1.8	7	6.8	1.2	0.49–2.5	4	6	0.76	0.20–1.87	57	68	0.84	0.64–1.09
Stomach	13	11.3	1.2	0.61–2.0	16	14.1	1.1	0.65–1.8	13	13	0.97	0.52–1.66	164	152	1.08	0.92–1.24
Radiation related sites *									68	147	0.47	0.37–0.60	1512	1804	0.84	0.80–0.88
Female breast	7	9.6	0.36	0.14–0.73	25	34.4	0.73	0.47–1.1								
Lung	22	23,3	0.94	0.59–1.4	35	31.6	1.1	0.77–1.5								
Colon	7	9,7	0.72	0.29–1.5	9	12.9	0.70	0.32–1.3								

* Includes cancer of the oesophagus, stomach. colon, liver, lung, female breast, ovary, bladder, non-melanoma of the skin, brain and nervous system, thyroid, bone and leukaemia.
